# Relationship between Virulence and Resistance among Gram-Negative Bacteria

**DOI:** 10.3390/antibiotics9100719

**Published:** 2020-10-20

**Authors:** Virginio Cepas, Sara M. Soto

**Affiliations:** ISGlobal, Hospital Clínic—Universitat de Barcelona, 08036 Barcelona, Spain; virginio.cepas@isglobal.org

**Keywords:** antimicrobial resistance, virulence, biofilms, Gram-negative: plasmids

## Abstract

Bacteria present in the human body are innocuous, providing beneficial functions, some of which are necessary for correct body function. However, other bacteria are able to colonize, invade, and cause damage to different tissues, and these are categorised as pathogens. These pathogenic bacteria possess several factors that enable them to be more virulent and cause infection. Bacteria have a great capacity to adapt to different niches and environmental conditions (presence of antibiotics, iron depletion, etc.). Antibiotic pressure has favoured the emergence and spread of antibiotic-resistant bacteria worldwide. Several studies have reported the presence of a relationship (both positive and negative, and both direct and indirect) between antimicrobial resistance and virulence among bacterial pathogens. This review studies the relationship among the most important Gram-negative bacteria (*Escherichia coli* and *Pseudomonas aeruginosa*) taking into account two points of view: (i) the effect the acquisition of resistance has on virulence, and (ii) co-selection of resistance and virulence. The relationship between resistance and virulence among bacteria depends on the bacterial species, the specific mechanisms of resistance and virulence, the ecological niche, and the host.

## 1. Introduction

Bacteria present in the human body are innocuous, providing beneficial functions, some of which are necessary for correct body function. However, other bacteria are able to colonise, invade and cause damage to different tissues and are categorized as pathogens. Pathogenic bacteria possess a variety of factors that allow them to cause damage to the host, but they are able to modify their virulence to adapt to the host immune system [1]. However, bacterial pathogenicity is a complex and multifactorial process, which depends on both human and bacterial factors, such as the host immune status and the bacterial virulence. The virulence mechanisms used by the bacteria to cause infection include adhesins, invasins, type three secretion systems, outer membrane proteins, toxins, capsules, iron acquisition systems, and biofilm formation, among others. Some of these mechanisms are chromosomic located such as some adhesins, but others are located within mobile genetic elements such as plasmids.

Nowadays, one of the most important health problems is the increasing emergence and spread of antimicrobial resistance among different microorganisms (bacteria, fungi, virus, and parasites). Among bacteria, resistance to antibiotics is rising in both community and hospital settings in association with an increase in mortality and morbidity. Antibiotic-resistant bacteria are estimated to cause 33,000 deaths in Europe and 700,000 in the world each year. In the worst-case scenario, by 2050, 10 million people in the world will die every year from bacterial infections, exceeding 1.8 million deaths from cancer [2]. The emergence of resistance in bacteria is threatening our ability to treat common infectious diseases, resulting in prolonged illness, disability, and death. Consequently, antimicrobial resistance increases healthcare costs, with lengthier hospital stays estimated at 2.5 million days of extra hospitalisation per year throughout the European Union, for a cost of about 900 million euros [3]. This poses serious challenges to the functioning of healthcare systems and represents high economic costs to society.

The development of new antibiotics with new mechanisms of action slowed in 1968 after the discovery of cephalosporins [4]. Most of the antibiotics developed thereafter are derivatives of the existing classes, being considered as “new-generation”. Unfortunately, the development of an antibiotic has, sooner or later, been followed by the emergence of bacterial strains resistant to these antibiotics [5]. There are three types of antibiotic resistance: (i) innate resistance by genes encoding inherent antibiotic resistance present in the bacteria, (ii) acquired resistance due to selective antibiotic pressure from the environment, and (iii) adaptative resistance that is a reflection of the ecological niche of the bacteria and includes environmentally induced genetic changes [6]. One of the most important mechanisms of the spread of antimicrobial resistance is through horizontal gene transfer (HGT). Thus, most acquired resistance is propagated by three ways of horizontal transfer between bacteria belonging to the same or different species or genera: mobile genetic elements (plasmids, transposons, etc.), transfection by bacteriophages, or by the acquisition of environmental DNA [7]. 

Although virulence and resistance are developed at different times (virulence from the beginning of host colonisation and resistance from the appearance of antibiotics), they are not independent characteristics but rather there is a relationship between them that depends on the bacterial species, the specific mechanisms of resistance and virulence, the ecological niche and environmental conditions, and the immune system of the host. In addition to the immune status, diet, age, and stress determine the host susceptibility to infection. Thus, opportunistic pathogens are able to cause infection in immunocompromised patients but not in healthy individuals [1].

Three scenarios have been reported: (i) an increase of resistance accompanied by an increase of virulence; (ii) an increase of resistance accompanied by a decrease of virulence; and (iii) an increase of resistance that does not cause effects on virulence.

In this work, this relationship among the most important Gram-negative bacteria (*Escherichia coli* and *Pseudomonas aeruginosa*) is revised taking into account two points of view: (i) the effect the acquisition of resistance has on virulence and (ii) the co-selection of resistance and virulence. 

## 2. Relationship between Antibiotic Resistance and Virulence in *Escherichia coli*

*Escherichia coli* is a well characterised microorganism frequently used as a laboratory model and in industrial microbiology [8]. In the human body, most gut-resident *E. coli* prevent colonisation by pathogenic bacteria and favour the host by producing vitamin K and B12, which are essential during the blood coagulation process and the formation of red blood cells, respectively [9,10,11]. However, some *E. coli* can also cause intestinal or extraintestinal infections such as urinary tract infections (UTIs), meningitis, and neonatal sepsis [12,13,14,15,16]. These pathogenic strains possess several virulence factors that allow them to colonise, invade, and cause damage to the host.

One of the relationships that has been described in different studies is that the acquisition of resistance to certain antimicrobial agents may be associated with a decrease in the virulence levels of a microorganism. The clearest example of this is found in uropathogenic strains of *E. coli* (UPEC). These strains possess certain virulence factors that allow them to colonize the urinary tract and produce cystitis (CT), pyelonephritis (PNP) and prostatitis (PT), and they can even reach the blood stream [17]. Many of these virulence factors, such as hemolysin, cytotoxic necrotizing factor, P-fimbriae, siderophore systems, S-fimbriae, yersiniabactin, toxin autotransporters, among others, are in regions of the chromosome called pathogenicity islands (PAIs) [18]. In the last decades, an increase in the level of resistance to antimicrobials such as quinolones in these microorganisms has been observed worldwide, possibly due to the fact they are considered the treatment of choice for UTIs. Studies among UPEC strains causing CT, PNP, or PT have been performed to establish the pattern of resistance, virulence, and the possibility of determining a relationship between these two features.

The first studies carried out on the virulence and resistance among UPEC strains causing CT and PNP by Bagel et al. 1999 [19] and Vila et al. 2002 [20] demonstrated that strains resistant to quinolones presented a decrease in the expression of type 1 fimbriae in comparison with susceptible strains (*p* = 0.019). In addition, a higher percentage of hemolysin-producing strains was found in strains susceptible to quinolones, and the presence of the *cnf1* and *sat* genes (encoding the cytotoxic necrotizing factor and toxin autotransporter, respectively) in these strains, both of which are involved in virulence, was also more frequent [20]. This study showed that quinolone resistant UPEC have fewer virulence factors, and they also show a decrease in the expression of type 1 fimbriae. Virulence factors located in the chromosome, such as aerobactin and P-fimbriae, were often absent in quinolone-resistant *E. coli* isolates [21,22,23]. 

A possible explanation for these results could be: when the strain is resistant to quinolones by a mutation in codon 83 of the DNA-gyrase, this mutation produces a reduction of the degree of supercoiling of the catalyzed DNA by this enzyme [19]. The expression of some genes has been linked to a negative supercoil, as is the case with *fimA*, which encodes type 1 fimbriae. To support this hypothesis, the role of the acquisition of a mutation in the *gyrA* gene in the virulence and protein expression of UPEC was studied. The HC14366M strain carrying a mutation in the *gyrA* gene (S83L) was found to lose the capacity to cause CT and PNP mainly due to a decrease in the expression of the *fimA*, *papA*, *papB*, and *ompA* genes. The levels of expression of the *fimA*, *papB*, and *ompA* genes were recovered on complementing the strain with a plasmid containing the *gyrA* wild-type gene. However, only a slight recovery was observed in the colonisation of the bladder in the GyrA complement strain compared to the mutant strain in a murine model of ascending UTI. Therefore, a mutation in the *gyrA* gene of UPEC reduced the virulence of the bacteria, likely in association with the effect of DNA supercoiling on the expression of several virulence factors and proteins, thereby decreasing their capacity to cause CT and PNP [24].

The second hypothesis is that, during the development of quinolone resistance, probably facilitated by exposure to these antimicrobials, these agents can act by enhancing the increase of the deletion and transposition of DNA regions (such as PAIs). These PAIs share some characteristics of bacteriophages, and it has been shown that prophages inserted into the chromosome are split by SOS system activity (see Section 5).

Nonetheless, one hypothesis does not preclude the other, and both mechanisms can be observed concomitantly.

Resistance to ciprofloxacin due to mutations in the *parC* and *gyrA* genes showed no reduction in in vitro growth rates. The biological cost of quinolone resistance differs among bacterial species and depends on the level of resistance and the number of resistance mutations, and highly resistant mutants with multiple mutations show significantly lower fitness than wild-type strains [25].

Several studies have determined that hemolysin-producing *E. coli* isolates were less resistant to tetracycline, nalidixic acid, cefotaxime and cotrimoxazol than non-hemolytic isolates [26,27]. In addition, there is a significant correlation between resistance to ceftazidime and cefotaxime with the presence of *fyuA* and *iutA* iron genes in UPEC isolates, while isolates harbouring the *chuA* genes were more susceptible to sulfamethoxazole [27].

However, a positive relationship has been found between the acquisition of resistance and virulence in the case of other antimicrobial agents. UPEC strains carrying the bla_CTX-M15_ presented more *colV*, *colE2-E9*, *colIa-Ib*, *hlyA*, and *csgA* genes. On the other hand, some UPEC strains present *bla*_OXA-2_ beta lactamase together with presence of *colM, colB, colE*, and *crl* genes [28]. It has been reported that *tetA* and *tetB* positive *E. coli* strains more often carry the virulence factors for P- fimbriae and aerobactin than susceptible strains [29].

Another mechanism that is involved in the dissemination of virulence and resistance is the presence of prophages. Thus, the Shiga toxin of *E. coli* is a virulence factor carried by prophages. These prophages can spread this toxin to other species that possess resistance mechanisms favouring the persistence or dissemination of both virulence and resistance in the environment [1].

Porins are barrel membranes that cross cell membranes and act as a pore through which molecules such as nutrients, toxins and antibiotics can diffuse. They play an important role in virulence and resistance, controlling not only the entry of antibiotics into the bacteria, but also the virulence factors [1]. In *E. coli*, OmpC is related to both characteristics. It has been observed that a deletion of this porin causes antibiotic resistance and a reduction of antibody-dependent bactericidal activity [30]. This porin has also been related to adhesion, cell invasion, and intestinal colonisation in patients with Crohn’s disease [31] (Table 1).

Therefore, positive and negative relationships between antibiotic resistance and virulence exist, depending on the antibiotic studied, the mechanism of resistance and the type of *E. coli*.

## 3. Relationship between Antibiotic Resistance and Virulence in *Pseudomonas aeruginosa*

*Pseudomonas aeruginosa* is an opportunistic human pathogen able to adapt to diverse natural and nosocomial conditions. This microorganism can cause pneumonia and bacteremia in immunocompromised hosts and is responsible for chronic lung disease in patients with cystic fibrosis. The prevalence of this pathogen in hospitals is due to its low susceptibility to antibiotics.

*P. aeruginosa* exhibits a wide range of virulence factors including elastase, proteases, phospholipase C, exotoxin A, exoenzyme S, pyocianine, rhamnolipids, lipopolysaccharide, flagella, and pili [32]. There is a correlation between a deficiency in virulence factor production and a decrease of susceptibility to antibiotics. Thus, *P. aeruginosa* isolates lacking elastase are more resistant to piperacillin, and no-pyocianine producing isolates are more resistant to amikacin, tobramycin, ciprofloxacin, and ceftazidime [32].

It has been observed that *P. aeruginosa* showing increased virulence related to the development of swarming motility presents greater resistance to polymyxin B, ciprofloxacin, and gentamicin [1]. This process involves the overexpression of different virulence factors such as the type III secretion system (TTSS), and proteases, among others.

Efflux pumps are involved in intrinsic and acquired resistance to antibiotics as well as in virulence, helping colonisation and growth on surfaces. Among these efflux pumps, those belonging to the resistance-nodulation-division family, play an important role in the pathogenesis of some bacteria and are involved in processes such as colonisation, infection and persistence [1]. One of the efflux pumps well-known in *P. aeruginosa* is the MexAB-OprM which is able to export quinolones, tetracyclines, chloramphenicol and beta lactams [33]. It has been reported that an increase in MexAB-OprD expression decreases the production of proteases and virulence [34]. In addition, the MuxABC-Omp efflux pump has been associated with macrolide resistance and virulence in *P. aeruginosa*. Thus, a mutant defective in the *muxABC-ompB* operon showed attenuated virulence due to a decrease in twitching motility but an increase in ampicillin and carbenicillin resistance. Other efflux pumps related to virulence and resistance are MexCD-OprJ and the MexEF-OprN. Overexpression of the MexCD-OprJ efflux pump causes a reduction in TTSS expression [35]. On the other hand, the overexpression of MexEF-OprN due to a mutation in the *nfxC* gene causes a decrease in the production of diverse virulence factors such as pyocianine, elastase, and rhamnolipids [34]. Finally, deletion of OprD, a channel for the uptake of basic amino acids, small peptides and carbapenem, led to an increase in bacterial fitness and virulence in a mouse infection model, as well as an increase in colonisation and systemic dissemination, and an increase in killing by human serum and low pH [36,37] (Table 2).

Mutations in the *gyrA* and *parC* genes conferring quinolone resistance in *P. aeruginosa* have been associated with a higher expression of the TTSS genes including the *exoS*, *exoT*, *exoU* and *exoY* genes involved in virulence. ExoS is a major cytotoxin involved in colonisation, invasion and dissemination of bacteria during infection [38]. ExoU has been related to diverse infections and to the severity of the infection [38]. Thus, *exoS+/exoU+ P. aeruginosa* strains have increased cytotoxicity and quinolone resistance [39].

Among *P. aeruginosa*, both positive and negative relationships between virulence and resistance can be found, playing an important role the levels of efflux pumps expression on the presence of secreted virulence factors.

## 4. Bacterial Biofilm and Its Relationship with Virulence and Resistance

Biofilms are well-organised 3D communities in which microorganisms live embedded, forming aggregates in self-produced extracellular polymeric substances (EPS), adhered to biological or inherent surfaces [40]. Biofilm communities confer special properties not present in free-living cells (Table 3) [41,42,43,44].

In many cases, biofilms worsen the course of infection since they increase antimicrobial treatment tolerance and trigger treatment failure (J William Costerton et al. 1995; Stewart 2015). Indeed, biofilms increase treatment resistance by up to 100–1000-fold compared to their planktonic counterparts [57]. Moreover, biofilms avoid innate and adaptive immune defences [58]. The biofilm architecture confers protection from external aggressions (i.e., pH and temperature changes, ultraviolet radiation, dryness, oxidation, metal ions or biocides), providing an ideal environment for their establishment [59,60,61,62].

This biofilm lifestyle provides a highly versatile structure which, unfortunately, makes treatment and eradication difficult. There is, therefore, an urgent need to increase our current knowledge about the genetics and physiological mechanisms of biofilms.

The mechanisms involved in biofilm antimicrobial tolerance can be classified into two groups (Table 4):

- Innate mechanisms, as a result of biofilm growth, such as extracellular matrix (ECM), metabolism heterogeneity and persister cells. Escape from the immune system can indirectly be included in this group, since the ECM provides cellular protection and allows persister cells to remain undetected by the host immune system.
- Induced or acquired resistance, derived from response to antimicrobial treatment (efflux pumps) or HGT, respectively.

Thus, innate mechanisms have been identified as factors that lead to biofilm antimicrobial tolerance, while induced and acquired resistance may contribute to biofilm antimicrobial tolerance but are not the main factors of biofilm recalcitrance [57,63]. Cell density and intercellular proximity inside biofilm promotes HGT through conjugation, transduction, and transformation, promoting both biofilm formation and gene transfer between biofilm forming species [54]. In this horizontal transmission, microorganisms harbouring plasmids containing antimicrobial resistance or virulence genes can contact other non-resistant cells, being able to transfer these plasmids and leading to new resistant or virulent cells [6].

Biofilms also play an important role in virulence, favouring the persistence and chronicity of infection as well as acting as a source of bacterial dissemination. Therefore, biofilm development is an essential part of bacterial pathogenicity, and it presents complex transcriptional regulation [6]. Within the biofilm, virulence genes can be activated through different mechanisms such as quorum sensing or two-component systems.

Apart that biofilm confer to the bacteria protection against the antibiotics and the immune system as well as virulence characteristics, a relationship between resistance and virulence exists within the formation of a biofilm structure. Thus, this structure facilitates the transference and the spread of antibiotic resistance and virulence genes among the cells forming the biofilm through HGT, among other mechanisms.

Several studies have demonstrated a relationship between biofilm formation and quinolone resistance in *E. coli*. Thus, among UPEC isolates, strains forming biofilm were significantly less resistant to nalidixic acid than those negative for biofilm formation (19% vs. 38%, *p* = 0.01) [64]. Salicylate induces a number of morphological and physiological alterations in bacteria including the activation of the transcriptional regulator MarA, which is also involved in quinolone resistance. An inverse relationship was observed between in vitro biofilm formation and the salicylate concentration added to the culture medium. Thus, the presence of salicylate increases the expression of *marA* and decreases the expression of the *fimA* and *fimB* genes in the wild-type strain. Therefore, the expression of type 1 fimbriae in the presence of salicylate may be regulated by the level of *marA* expression through a *fimB* regulator, albeit through neither the *ompX* nor the *tolC* genes [65].

On the other hand, a positive relationship has been found between *P. aeruginosa* resistant strains and biofilm formation, with resistant strains presenting higher biofilm production than susceptible strains [66].

## 5. Co-Selection of Resistance and Virulence

In addition to the effects that the acquisition of antibiotic resistance has on virulence, a co-selection of both characteristic can occur through mobile genetic elements such as plasmids and integrative and conjugative elements (integrative conjugative elements, ICEs: transposons, PAIs, integrons, etc.). These elements are transferred among bacterial strains belonging to the same or different species through three different mechanisms: conjugation, transformation, and transduction.

Plasmids are extra-chromosomal and self-replicating elements that are not essential to bacteria but which often carry and spread genes that confer the bacteria characteristics such as virulence, resistance, and persistence that allow them to adapt to different environments and niches [67]. Conjugative plasmids play an important role in the dissemination of resistance and virulence through HGT, with those belonging to the IncF incompatibility group being the most involved in this process. For example, the *E. coli* ST131 clone expresses the beta lactamase CTX-M15 that is located in a plasmid and improves the persistence of this bacteria during infection.

The dissemination of conjugative plasmids carrying both virulence and resistance determinants leads to a simultaneous selection of both types of factors in an effective environment with or without antibiotic pressure [1]. The spread of these plasmids among the most pathogenic bacteria can be an important health problem.

ICEs are also self-transmissible mobile genetic elements that disseminate genes located in another higher DNA molecule such as chromosomes and plasmids through HGT.

Among these ICEs, we found PAIs that represent distinct large chromosomal regions that contribute to the evolution of bacterial pathogens [68] and carry virulence genes. In vitro studies with UPEC isolates showed that antimicrobials and, in particular, quinolones, induce the loss of virulence factors located in certain PAIs in a SOS-dependent or –independent manner. They can also induce mutations in punctual genes that prevent exit outside the cells of toxins or modify the functionality of certain virulence factors, such as hemolysin [68]. In addition, UPEC strains submitted to subinhibitory concentrations of ciprofloxacin lost the PAI-I and the ability to colonise the kidneys in a mouse model of urine infection due to a decrease in *papA* gene expression (Soto-unpublished data)

This relationship has been demonstrated in vivo by studying *E. coli* strains isolated from patients receiving treatment with quinolones [69]. In this study, 30 *E. coli* isolates were collected from 15 patients with a febrile UTI presenting bacteriological recurrence during long-term follow-up. Horizontal transfer of virulence factors contained in a PAI had occurred in one isolate after 14 days of treatment with ciprofloxacin [69]. Quinolone resistance is not necessary for *E. coli* strains to lose PAIs; however, quinolone-resistant *E. coli* strains have likely been in previous contact with these antimicrobial agents, thus favouring the loss of the PAI (Table 5).

## 6. Conclusions

Antimicrobial resistance and virulence are not two independent characteristics but rather a negative or positive relationship can be found between them. This relationship offers advantages to the microorganisms, conferring characteristics that allow them to survive in different niches with different selective pressures (presence of antibiotics, etc.). Thus, the acquisition of antibiotic resistance can induce the loss of virulence factors and biofilm formation among UPEC strains but, in other cases, an increase in antibiotic resistance enhances virulence capacity. In some cases, this relationship is very direct. When the virulent pathogen is present in the host causing infection, antimicrobial treatment is given, that could be associated to an increase of bacterial resistance. In addition, mobile genetic elements are an important route of dissemination of both virulence and resistance and could be an important health problem due to the acquisition of these mobile genetic elements carrying both virulence and resistance genes, could become susceptible strains to more pathogenic and resistant bacteria. Therefore, not only are new antibiotics needed, but anti-virulence molecules may also be useful in the fight against the emergence and dissemination of antibiotic-resistant strains.

## Figures and Tables

**Table 1:** Relationship resistance-virulence among *E. coli* explained in this review.

Resistance	Involved Mechanism	Effect on Virulence
**Quinolones**	*gyrA* gene mutation	Decreased expression of type 1 fimbriae, P-fimbriae and OmpA. Absence of *cnf1*, *hlyA* and *sat* genes.
**Quinolones**	SOS-system activation	Loss of virulence genes located into PAIs.
**Betalactamics**	Presence of *bla*_CTx-M-15_ gene	High presence of *colV*, *colE2-E9*, *colIa-Ib*, *hlyA*, *csgA* genes.
**Betalactamics**	Presence of *bla*_OXA-2_ gene	High presence of *colM*, *colB*, *colE*, *crl* genes.
**Tatracyclines**	Presence of *tetA* and *tetB* genes	Increased expression of P-fimbriae and aerobactin.
**Antibiotics**	OmpC deletion	Decreased antibody-dependent bactericidal activity, adhesion, invasion and intestinal colonisation.

**Table 2:** Relationship resistance-virulence among *P. aeruginosa* explained in this review.

Resistance	Effect on Virulence
**Piperacillin**	No elastase production.
**Amikacin, Tobramycin, Ciprofloxacin, Ceftazidime**	No pyocianine production.
**Quinolones by *gyrA*-*parC* mutations**	Overexpression of TTSS (*exoU, exoS, exoT* and *exoY* genes)
**Overexpression MexAB-OprD**	Low expression of proteases and virulence.
**Deletion MuxABC-OprB**	Decreased twitching motility.
**Overexpression MexCD-OprJ**	Decreased TTSS expression.
**Overexpression MexEF-OprJ**	Decreased production of pyocianine, elastases, and rhamnolipids.
**Deletion of OprD**	Decreased bacterial fitness. Increased colonisation and systemic infection.

**Table 3:** Biofilm mechanisms for adaptation to the media.

Property	Advantages	References
Localised gradients of nutrients, oxygen, pH, and quorum sensing molecules	As a result of biofilm heterogeneity, gradients are generated which provide multiple habitats in which microbial cells can establish, depending on their physiological requirements.	[45]
Tolerance to desiccation	EPS confer structural protection against dehydrated environments. Studies in bacterial biofilm confirm that bacteria overproduce EPS molecules in dry environments.	[46,47]
Intraspecies cooperation and metabolic cooperativity	Biofilms favour intraspecies cooperation producing microenvironments that favour growth conditions. For example, nutrient cycling (carbon, nitrogen, sulphur) confers new nutrient sources inside the biofilm.	[48]
Antimicrobial tolerance	Biofilm lifestyle allows microorganisms to develop tolerance to antimicrobial therapies. Matrix can hinder diffusion or inactivate antimicrobial agents.	[42,49,50]
Persister cells and dormant cells	In these stages microbial cells remain inside biofilms and lead to treatment failure.	[51]
Efflux pumps	Efflux pumps promote antifungal and antibiotic depletion from the biofilm.	[52,53]
Exchange of genetic material	Cell-to-cell contact improves horizontal gene transfer given the cell-to-cell contact environment.	[54]
Enzyme degradation	Biofilm accumulates derived and waste products from metabolic processes. Enzymatic activity can recycle these nonviable subtracts to viable nutrients and degradation of EPS for cell dispersal.	[45,55]
Sorption	The sorption effect provides nutrients, gases and other molecule exchange.	[56]

**Table 4:** Relationship between virulence and resistance among biofilms.

Type of Resistance	Mechanisms of Resistance	Effect on Virulence
Innate	Extracellular matrix (ECM), metabolism heterogeneity and persister cells.	Antibiotic treatment and immune system protection.
Induced or Acquired	Efflux pumps, horizontal gene transference (HGT)	Transference of plasmids harbouring resistance and virulence determinants

**Table 5:** Relationships among resistance and virulence through mobile genetic elements.

Mobile Genetic Elements	Effect on Virulence
Conjugative plasmids	Transference of virulence and resistance determinants intra- and interspecies.
ICEs (transposons, PAIS, etc.)	Loss of PAIs due to exposure to quinolones in UPEC.

## References

[1] Beceiro A., Tomás M., Bou G. (2013). Antimicrobial Resistance and Virulence: A Successful or Deleterious Association in the Bacterial World?. Clin. Microbiol. Rev..

[2] Glasner C., Albiger B., Buist G., Andrašević A.T., Cantón R., Carmeli Y., Friedrich A.W., Giske C.G., Glupczynski Y., Gniadkowski M. (2013). Carbapenemase-producing Enterobacteriaceae in Europe: A survey among national experts from 39 countries, February 2013. Eurosurveillance.

[3] Powers J. (2004). Antimicrobial drug development – the past, the present, and the future. Clin. Microbiol. Infect..

[4] Saga T., Yamaguchi K. (2009). History of antimicrobial agents and resistant bacteria. Jpn. Med. Assoc. J..

[5] Moskowitz S.M., Foster J.M., Emerson J., Burns J.L. (2004). Clinically Feasible Biofilm Susceptibility Assay for Isolates of Pseudomonas aeruginosa from Patients with Cystic Fibrosis. J. Clin. Microbiol..

[6] Schroeder M., Brooks B.D., Brooks A.E. (2017). The Complex Relationship between Virulence and Antibiotic Resistance. Genes.

[7] Von Wintersdorff C.J.H., Ependers J., Van Niekerk J.M., Mills N.D., Emajumder S., Van Alphen L.B., Savelkoul P.H.M., Wolffs P.F.G. (2016). Dissemination of Antimicrobial Resistance in Microbial Ecosystems through Horizontal Gene Transfer. Front. Microbiol..

[8] Blount Z.D. (2015). The unexhausted potential of *E. coli*. eLife.

[9] Bentley R., Meganathan R. (1982). Biosynthesis of vitamin K (menaquinone) in bacteria. Microbiol. Rev..

[10] Lawrence J.G., Roth J.R. (1996). Evolution of Coenzyme B(12) Synthesis among Enteric Bacteria: Evidence for Loss and Reacquisition of a Multigene Complex. Genetics.

[11] Eggesbø M., Moen B., Peddada S.D., Baird D.D., Rugtveit J., Midtvedt T., Bushel P.R., Sekelja M., Rudi K. (2010). Development of gut microbiota in infants not exposed to medical interventions. APMIS.

[12] Pratt L.A., Kolter R. (1998). Genetic analysis of *Escherichia coli* biofilm formation: Roles of flagella, motility, chemotaxis and type I pili. Mol. Microbiol..

[13] Soto S.M. (2014). Importance of Biofilms in Urinary Tract Infections: New Therapeutic Approaches. Adv. Biol..

[14] Millán-Rodríguez F., Palou J., Bujons-Tur A., Musquera-Felip M., Sevilla-Cecilia C., Serrallach-Orejas M., Baez-Angles C., Villavicencio-Mavrich H. (2005). Acute bacterial prostatitis: Two different sub-categories according to a previous manipulation of the lower urinary tract. World J. Urol..

[15] Beloin C., Roux A., Ghigo J.M. (2008). Escherichia coli biofilms. Curr. Top Microbiol. Immunol..

[16] Wright K.J., Seed P.C., Hultgren S.J. (2005). Uropathogenic *Escherichia coli* Flagella Aid in Efficient Urinary Tract Colonization. Infect. Immun..

[17] Pompilio A., Crocetta V., Savini V., Petrelli D., Di Nicola M., Bucco S., Amoroso L., Bonomini M., Di Bonaventura G. (2018). Phylogenetic relationships, biofilm formation, motility, antibiotic resistance and extended virulence genotypes among *Escherichia coli* strains from women with community-onset primitive acute pyelonephritis. PLoS ONE.

[18] Hacker J., Blum-Oehler G., Muhldorfer I., Tschape H. (1997). Pathogenicity islands of virulent bacteria: Structure, function and impact on microbial evolution. Mol. Microbiol..

[19] Bagel S., Hüllen V., Wiedemann B., Heisig P. (1999). Impact of *gyrA* and *parC*Mutations on Quinolone Resistance, Doubling Time, and Supercoiling Degree of *Escherichia coli*. Antimicrob. Agents Chemother..

[20] Vila J., Simon K., Ruiz J., Horcajada J.P., Velasco M., Barranco M., Moreno A., Mensa J. (2002). Are Quinolone-Resistant Uropathogenic *Escherichia coli* Less Virulent?. J. Infect. Dis..

[21] Johnson J.R., Moseley S.L., Roberts P.L., E Stamm W. (1988). Aerobactin and other virulence factor genes among strains of *Escherichia coli* causing urosepsis: Association with patient characteristics. Infect. Immun..

[22] Horcajada J.P., Soto S.M., Gajewski A., Smithson A., De Anta M.T.J., Mensa J., Vila J., Johnson J.R. (2005). Quinolone-Resistant Uropathogenic *Escherichia coli* Strains from Phylogenetic Group B2 Have Fewer Virulence Factors than Their Susceptible Counterparts. J. Clin. Microbiol..

[23] Liu X., Liu H., Li Y., Hao C. (2017). Association between virulence profile and fluoroquinolone resistance in *Escherichia coli* isolated from dogs and cats in China. J. Infect. Dev. Ctries..

[24] Sánchez-Céspedes J., Sáez-López E., Frimodt-Møller N., Vila J., Soto S.M. (2015). Effects of a Mutation in the gyrA Gene on the Virulence of Uropathogenic Escherichia coli. Antimicrob. Agents Chemother..

[25] Kishii R., Takei M. (2009). Relationship between the expression of ompF and quinolone resistance in Escherichia coli. J. Infect. Chemother..

[26] Tabasi M., Karam M.R.A., Habibi M., Yekaninejad M.S., Bouzari S. (2015). Phenotypic Assays to Determine Virulence Factors of Uropathogenic *Escherichia coli* (UPEC) Isolates and their Correlation with Antibiotic Resistance Pattern. Osong Public Health Res. Perspect..

[27] Karam M.R.A., Habibi M., Bouzari S. (2018). Relationships between Virulence Factors and Antimicrobial Resistance among *Escherichia coli* Isolated from Urinary Tract Infections and Commensal Isolates in Tehran, Iran. Osong Public Health Res. Perspect..

[28] El-Baky R.M.A., Ibrahim R.A., Mohamed D.S., Ahmed E.F., Hashem Z.S. (2020). Prevalence of Virulence Genes and Their Association with Antimicrobial Resistance Among Pathogenic *E. coli* Isolated from Egyptian Patients with Different Clinical Infections. Infect. Drug Resist..

[29] Karami N., Nowrouzian F.L., Adlerberth I., Wold A. (2006). Tetracycline Resistance in *Escherichia coli* and Persistence in the Infantile Colonic Microbiota. Antimicrob. Agents Chemother..

[30] Liu Y.-F., Yan J.-J., Lei H.-Y., Teng C.-H., Wang M.-C., Tseng C.-C., Wu J.-J. (2012). Loss of Outer Membrane Protein C in *Escherichia coli* Contributes to Both Antibiotic Resistance and Escaping Antibody-Dependent Bactericidal Activity. Infect. Immun..

[31] Rolhion N., Carvalho F.A., Darfeuille-Michaud A. (2007). OmpC and the sigmaE regulatory pathway are involved in adhesion and invasion of the Crohn’s disease-associated *Escherichia coli* strain LF82. Mol. Microbiol..

[32] Karatuna O., Yagci A. (2010). Analysis of quorum sensing-dependent virulence factor production and its relationship with antimicrobial susceptibility in Pseudomonas aeruginosa respiratory isolates. Clin. Microbiol. Infect..

[33] Poole K., Gotoh N., Tsujimoto H., Zhao Q., Wada A., Yamasaki T., Neshat S., Yamagishi J.-I., Li X.-Z., Nishino T. (1996). Overexpression of the mexC-mexD-oprJ efflux operon innfxB-type multidrug-resistant strains of Pseudomonas aeruginosa. Mol. Microbiol..

[34] Linares J.F., López J.A., Camafeita E., Albar J.P., Rojo F., Martinez J.L. (2005). Overexpression of the Multidrug Efflux Pumps MexCD-OprJ and MexEF-OprN Is Associated with a Reduction of Type III Secretion in Pseudomonas aeruginosa. J. Bacteriol..

[35] Geisinger E., Isberg R.R. (2017). Interplay Between Antibiotic Resistance and Virulence During Disease Promoted by Multidrug-Resistant Bacteria. J. Infect. Dis..

[36] Roux D., Danilchanka O., Guillard T., Cattoir V., Aschard H., Fu Y., Angoulvant F., Messika J., Ricard J.-D., Mekalanos J.J. (2015). Fitness cost of antibiotic susceptibility during bacterial infection. Sci. Transl. Med..

[37] Skurnik D., Roux D., Cattoir V., Danilchanka O., Lu X., Yoder-Himes D.R., Han K., Guillard T., Jiang D., Gaultier C. (2013). Enhanced in vivo fitness of carbapenem-resistant oprD mutants of Pseudomonas aeruginosa revealed through high-throughput sequencing. Proc. Natl. Acad. Sci. USA.

[38] Kulasekara B.R., Kulasekara H.D., Wolfgang M.C., Stevens L., Frank D.W., Lory S. (2006). Acquisition and Evolution of the exoU Locus in Pseudomonas aeruginosa. J. Bacteriol..

[39] Cho H.H., Kwon K.C., Kim S., Koo S.H. (2014). Correlation Between Virulence Genotype and Fluoroquinolone Resistance in Carbapenem-Resistant Pseudomonas aeruginosa. Ann. Lab. Med..

[40] Vert M., Doi Y., Hellwich K., Hess M., Hodge P., Kubisa P., Rinaudo M., Schué F. (2012). Terminology for biorelated polymers and applications. Pure Appl. Chem..

[41] Coenye T. (2010). Response of sessile cells to stress: From changes in gene expression to phenotypic adaptation. FEMS Immunol. Med. Microbiol..

[42] Costerton J.W., Stewart P.S., Greenberg E.P. (1999). Bacterial Biofilms: A Common Cause of Persistent Infections. Science.

[43] Konopka A. (2009). What is microbial community ecology?. ISME J..

[44] Lerch T.Z., Chenu C., Dignac M.F., Barriuso E., Mariotti A. (2017). Biofilm vs. Planktonic Lifestyle: Consequences for Pesticide 2,4-D Metabolism by Cupriavidus necator JMP134. Front. Microbiol..

[45] Flemming H.-C., Wingender J. (2010). The biofilm matrix. Nat. Rev. Genet..

[46] Ledwoch K., Maillard J.-Y. (2018). Candida auris Dry Surface Biofilm (DSB) for Disinfectant Efficacy Testing. Materials.

[47] Potts M. (1994). Desiccation tolerance of prokaryotes. Microbiol. Rev..

[48] Elias S., Banin E. (2012). Multi-species biofilms: Living with friendly neighbors. FEMS Microbiol. Rev..

[49] Gu J.D., Roman M., Esselman T., Mitchell R. (1998). The role of microbial biofilms in deterioration of space station candidate materials. Intern. Biodeter. Biodegrad..

[50] Mateus C., Crow S.A., Ahearn D.G. (2004). Adherence of Candida albicans to Silicone Induces Immediate Enhanced Tolerance to Fluconazole. Antimicrob. Agents Chemother..

[51] Fisher R.A., Gollan B., Helaine S. (2017). Persistent bacterial infections and persister cells. Nat. Rev. Genet..

[52] Nobile C.J., Johnson A.D. (2015). Candida albicans Biofilms and Human Disease. Annu. Rev. Microbiol..

[53] Stewart P.S. (2015). Antimicrobial Tolerance in Biofilms. Microbiol. Spectr..

[54] Madsen J.S., Burmølle M., Hansen L.H., Sørensen S.J. (2012). The interconnection between biofilm formation and horizontal gene transfer. FEMS Immunol. Med. Microbiol..

[55] Whitfield G.B., Marmont L.S., Howell P.L. (2015). Enzymatic modifications of exopolysaccharides enhance bacterial persistence. Front. Microbiol..

[56] Billings N., Birjiniuk A., Samad T.S., Doyle P.S., Ribbeck K. (2015). Material properties of biofilms—a review of methods for understanding permeability and mechanics. Rep. Prog. Phys..

[57] Høiby N., Bjarnsholt T., Givskov M., Molin S., Ciofu O. (2010). Antibiotic resistance of bacterial biofilms. Int. J. Antimicrob. Agents.

[58] Gonzalez J.F., Hahn M.M., Gunn J.S. (2018). Chronic biofilm-based infections: Skewing of the immune response. Pathog. Dis..

[59] Espeland E., Wetzel R. (2001). Complexation, Stabilization, and UV Photolysis of Extracellular and Surface-Bound Glucosidase and Alkaline Phosphatase: Implications for Biofilm Microbiota. Microb. Ecol..

[60] Le Magrex-Debar E., Lemoine J., Gellé M.-P., Jacquelin L.-F., Choisy C. (2000). Evaluation of biohazards in dehydrated biofilms on foodstuff packaging. Int. J. Food Microbiol..

[61] Teitzel G.M., Parsek M.R. (2003). Heavy Metal Resistance of Biofilm and Planktonic Pseudomonas aeruginosa. Appl. Environ. Microbiol..

[62] Welin-Neilands J., Svensäter G. (2007). Acid Tolerance of Biofilm Cells of Streptococcus mutans. Appl. Environ. Microbiol..

[63] Donlan R.M., Costerton J.W. (2002). Biofilms: Survival Mechanisms of Clinically Relevant Microorganisms. Clin. Microbiol. Rev..

[64] Soto S., Smithson A., Martinez J., Horcajada J., Mensa J., Vila J. (2007). Biofilm Formation in Uropathogenic *Escherichia coli* Strains: Relationship With Prostatitis, Urovirulence Factors and Antimicrobial Resistance. J. Urol..

[65] Vila J., Soto S.M. (2012). Salicylate increases the expression of marA and reduces in vitro biofilm formation in uropathogenic *Escherichia coli* by decreasing type 1 fimbriae expression. Virulence.

[66] Drenkard E., Ausubel F.M. (2002). Pseudomonas biofilm formation and antibiotic resistance are linked to phenotypic variation. Nat. Cell Biol..

[67] Johnson T.J., Nolan L.K. (2009). Pathogenomics of the Virulence Plasmids of Escherichia coli. Microbiol. Mol. Biol. Rev..

[68] Soto S.M., De Anta M.T.J., Vila J. (2006). Quinolones Induce Partial or Total Loss of Pathogenicity Islands in Uropathogenic *Escherichia coli* by SOS-Dependent or -Independent Pathways, Respectively. Antimicrob. Agents Chemother..

[69] Soto S.M., Zúñiga S., Ulleryd P., Vila J. (2011). Acquisition of a pathogenicity island in an *Escherichia coli* clinical isolate causing febrile urinary tract infection. Eur. J. Clin. Microbiol. Infect. Dis..

